# The frequency of promoter DNA hypermethylation is decreased in colorectal neoplasms of familial adenomatous polyposis

**DOI:** 10.18632/oncotarget.25987

**Published:** 2018-08-24

**Authors:** Kiyoko Takane, Masaki Fukuyo, Keisuke Matsusaka, Satoshi Ota, Bahityar Rahmutulla, Kazuyuki Matsushita, Hideaki Miyauchi, Yukio Nakatani, Hisahiro Matsubara, Atsushi Kaneda

**Affiliations:** ^1^ Department of Molecular Oncology, Graduate School of Medicine, Chiba University, Chiba, Japan; ^2^ Department of Genome Research and Development, Kazusa DNA Research Institute, Chiba, Japan; ^3^ Department of Pathology, Chiba University Hospital, Chiba, Japan; ^4^ Department of Laboratory Medicine and Division of Clinical Genetics and Proteomics, Chiba University Hospital, Chiba, Japan; ^5^ Department of Frontier Surgery, and Graduate School of Medicine, Chiba University, Chiba, Japan; ^6^ Department of Diagnostic Pathology, Graduate School of Medicine, Chiba University, Chiba, Japan

**Keywords:** familial adenomatous polyposis (FAP), colorectal cancer, colorectal adenoma, DNA methylation

## Abstract

Familial adenomatous polyposis (FAP) is an inherited disorder characterized by numerous colorectal adenomatous polyps with predisposition to the development of colorectal cancer (CRC). Here, we conducted genome-wide DNA methylation analysis of FAP neoplasms, including seven cancer samples and 16 adenoma samples, using an Infinium 450K BeadArray. As controls for sporadic colorectal neoplasms and mucosae, we used Infinium 450k data from 297 CRC samples, 45 colorectal adenoma samples, and 37 normal mucosa samples with reference to The Cancer Genome Atlas and other databases. Unsupervised two-way hierarchical clustering analysis of FAP and sporadic CRC/adenoma revealed that CRC was classified into four DNA methylation epigenotypes (MEs): high-ME (HME), intermediate-ME (IME), low-ME (LME), and normal-like ME (NME). Five FAP neoplasms (two cancer and three adenoma) were clustered with IME, whereas 18 FAP neoplasms (five cancer and 13 adenoma) were clustered into NME. IME FAP neoplasms significantly correlated with *KRAS* mutations, similar to sporadic CRC. Within IME cases, however, aberrant DNA methylation was significantly less frequent in FAP neoplasms than sporadic neoplasms, and these unmethylated genes included WNT family genes and several types of oncogenes. In summary, FAP neoplasms were classified into at least two molecular subtypes, i.e., NME in the majority of cases showing mostly no aberrant methylation and IME in some cases accompanied by *KRAS* mutations but less frequent aberrant DNA methylation than sporadic neoplasms, suggesting that FAP may follow a tumorigenesis pathway different from that of sporadic CRC.

## INTRODUCTION

Sporadic colorectal cancer (CRC) is a major cause of death and is associated with high incidence and mortality rates [1]. Sporadic CRC arises through accumulation of genetic alterations, called the “adenoma-carcinoma sequence”, which involves progression from adenomatous polyps to CRC [2–5]. In addition to genetic alterations due to chromosomal instability and microsatellite instability [6], aberrant DNA methylation of promoter CpG islands has been reported to be one of the most important epigenomic alterations in colorectal carcinogenesis, leading to inactivation of multiple tumor-suppressor genes [7].

In our previous study, we performed epigenotyping of sporadic CRC using comprehensive and quantitative DNA methylation data [8]. Although some genes were commonly hypermethylated in nearly all CRC cases, two groups of classifier genes (Group-1 and Group-2 markers) were established to classify sporadic CRC into three distinct DNA methylation epigenotypes (MEs): high-ME (HME), intermediate-ME (IME), and low-ME (LME) [8]. Group-1 markers are mostly equivalent to classical markers for the CpG island methylator phenotype (CIMP) [9]. HME/CIMP-high sporadic CRC showed hypermethylation of both Group-1 and Group-2 markers and was strongly correlated with *BRAF* mutations. IME/CIMP-low sporadic CRC showed methylation of Group-2, but not Group-1 markers, and was strongly correlated with *KRAS* mutations. Finally, LME sporadic CRC showed aberrant hypermethylation of commonly methylated genes, but no methylation of Group-1/Group-2 markers, and did not correlate with *BRAF* or *KRAS* mutations, as reported by other groups [9, 10].

Familial adenomatous polyposis (FAP) is an autosomal dominant inherited disorder characterized by numerous colorectal adenomatous polyps with predisposition to the development of CRC. The worldwide incidence of FAP is 3–10 per 100,000, accounting for approximately 1% of CRC cases [11]. In patient with FAP, the risk of progression to CRC by the age of 35–40 years is approximately 100% [12]. Despite this extremely high risk of cancer, the molecular basis of tumorigenesis in FAP is not fully understood. The “second hit” against *APC* has not been definitively identified in patients with FAP with germline mutations in *APC* [13]. Moreover, approximately 20% of patients with FAP do not possess *APC* germline mutations, and responsible mutations have not yet been identified [14]. With regard to epigenetic alterations, few studies have described aberrant DNA methylation with a focus on FAP, and molecular stratification of FAP neoplasms has yet to be clarified [15].

We recently evaluated DNA methylation epigenotypes of FAP neoplasms by pyrosequencing using 20 methylation marker genes, which we previously established [16]. There are thought to be at least two molecular subtypes of FAP neoplasms, and these lesions appear to be similar to LME and IME in sporadic CRC. The two epigenotypes are independent of *APC* germline mutation status, and both subtypes can develop malignant tumors. Comprehensive analysis of DNA methylation status other than the 20 methylation markers or investigation of differences between FAP and sporadic neoplasms, however, has not yet been clarified.

Therefore, in this study, we conducted genome-wide DNA methylation analysis of FAP neoplasms using an Infinium HumanMethylation450 BeadChip (Infinium 450k) array with reference to the Infinium 450k data of The Cancer Genome Atlas (TCGA) and other data [17]. Our findings provided important insights into the specific features of FAP-associated colorectal neoplasms.

## RESULTS

### Extraction of Infinium probes that were not significantly influenced by formalin fixation and paraffin embedding

**Figure 1:**
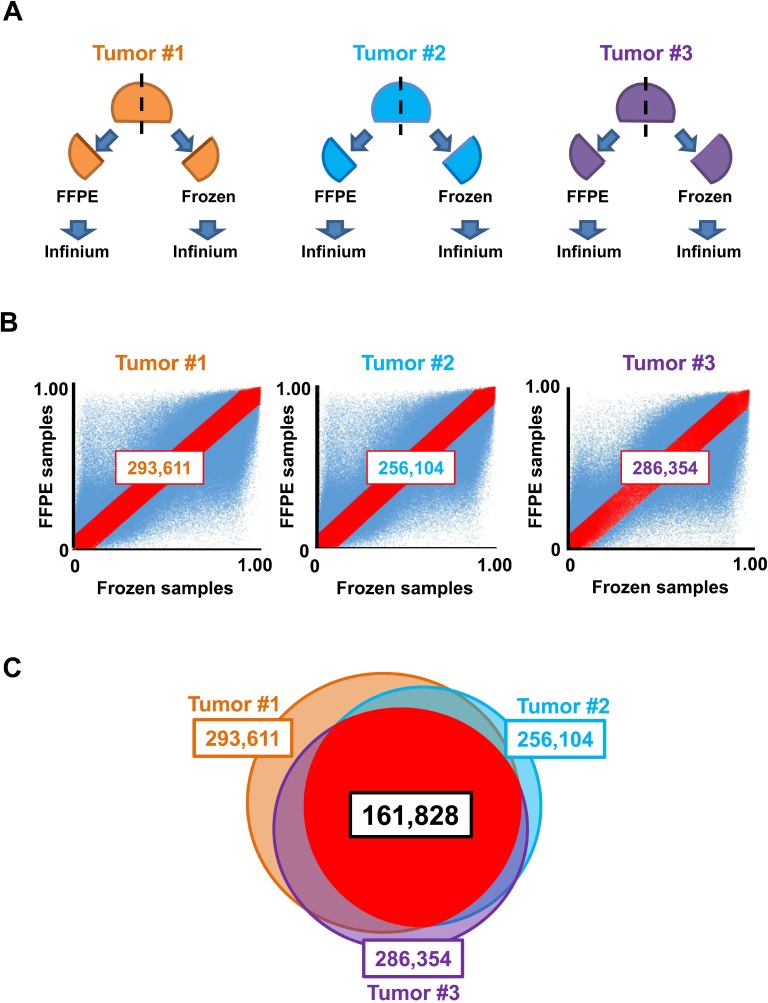
Selection of appropriate probes for analysis of frozen and FFPE samples (**A**) Preparation of frozen and FFPE samples. Three colon tumors (#1–3) were cut into two pieces; one was fixed with formalin and embedded in paraffin (*FFPE*), and the other was frozen with liquid nitrogen and stored at –80° C (*frozen*). Both tissues underwent DNA extraction and Infinium assays. (**B**) Plot of β-values. Probes showing the differences in β-values between frozen and FFPE samples of less than 0.1, i.e., between y = x + 0.1 and y = x−0.1, were extracted (*red*). (**C**) In total, 161,828 overlapped probes among three analyses (tumors #1, #2, and #3) were extracted and used for subsequent DNA methylation analysis.

DNA derived from formalin-fixed, paraffin-embedded (FFPE) tissue is generally of poor integrity due to DNA-protein crosslinks and nucleic acid fragmentation, rendering this source of DNA less compatible with Infinium [18]. Some protocols have been reported to improve quality of FFPE DNA samples and achieve robust array results [19, 20]. Because both frozen and FFPE samples were analyzed in this study, probes that were not significantly influenced by FFPE were extracted to exclude the effects of the FFPE procedure for Infinium analysis. Infinium 450k analysis was performed for three pairs of frozen and FFPE colorectal neoplastic samples (tumors #1, #2, and #3; Figure 1A). The numbers of probes with differences in β-values of less than 0.1 between frozen and FFPE samples were 293,611 (tumor #1), 256,104 (tumor #2), and 286,354 (tumor #3; Figure 1B). The number of probes with differences in β-values of less than 0.1 in all three neoplastic samples was 161,828, and we used these probes for subsequent DNA methylation analysis in frozen and FFPE samples (Figure 1C).

### Hierarchical clustering analysis of FAP neoplasms and sporadic CRC

**Figure 2:**
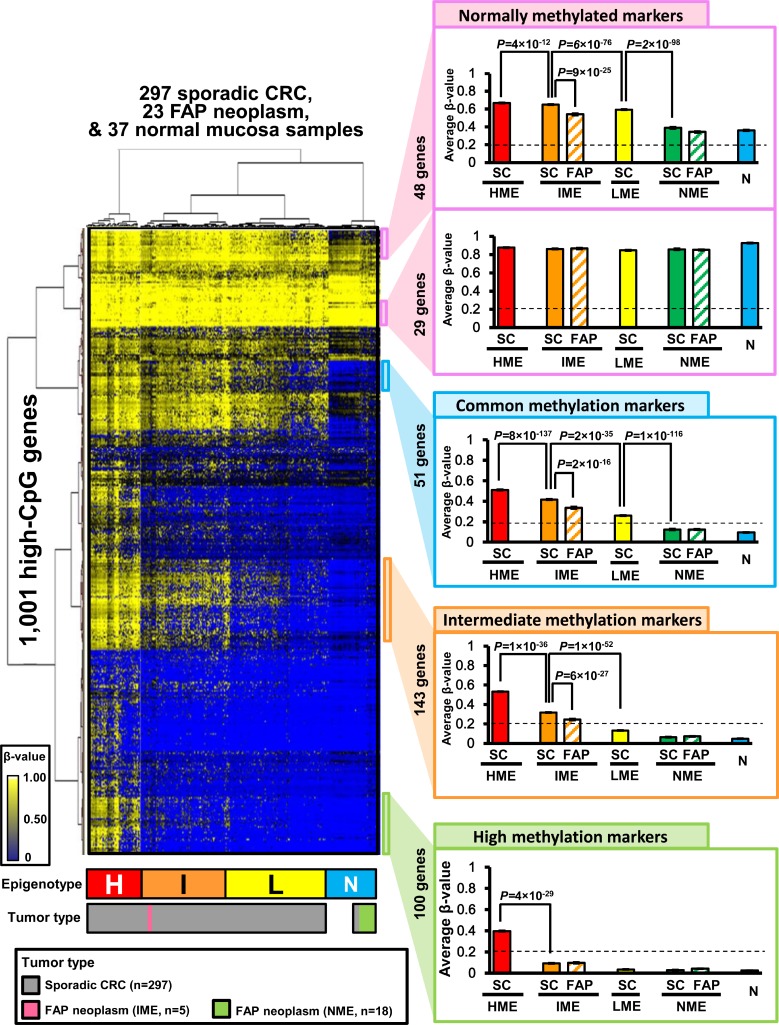
Hierarchical clustering analysis of FAP neoplasms, sporadic CRC, and normal mucosa samples Using unsupervised two-way hierarchical clustering analysis, 357 clinical samples were clustered into four methylation epigenotypes: high, intermediate, low, and normal-like methylation epigenotypes (*H*, HME; *I*, IME; *L*, LME; *N*, NME) (left panel). Methylation levels were compared among four epigenotypes using subgroups of genes (right panel). *SC*, sporadic CRC; *FAP*, FAP neoplasms; *N*, normal mucosa. Error bars show standard errors. Normally methylated markers are shown in the top panel, and commonly methylated markers are shown in the second from top panel. Intermediate methylation markers are shown in the third from top panel, and high methylation markers are shown in the bottom panel.

Next, we performed Infinium 450k analysis for 23 FAP neoplasm samples (Supplementary Table 1) and utilized β-values of the extracted 161,828 probes. We also analyzed Infinium 450 k data for 297 sporadic CRC and 37 normal mucosa samples supplied by TCGA. Among 161,828 probes, 49,551 probes were located in the promoter region of 8,625 high-CpG genes. When multiple probes were designed within a promoter, the probe nearest to the transcription start site was selected for each gene. Among these 8,625 probes (i.e., genes), 1,001 probes with standard deviation of β-values > 0.10 in 357 samples were selected. Unsupervised two-way hierarchical clustering analysis was performed using the β-values of these 1,001 probes in 23 FAP colorectal neoplasm samples, 297 sporadic CRC samples, and 37 normal colorectal mucosa samples (Figure 2). The 357 clinical samples were clearly clustered into four MEs, i.e., HME, IME, LME, and another ME. All 37 normal mucosa samples were clustered into the fourth ME, which was named normal-like ME (NME) hereafter, and the NME samples also included 10 (3%) of 297 sporadic CRC and 18 (78%) of 23 FAP neoplasm samples. Although sporadic CRC samples were distributed into four MEs, FAP neoplasms were classified into two MEs, i.e., IME (*n* = 5, 22%) and NME (*n* = 18, 78%).

Two-way hierarchical clustering analysis also stratified the 1,001 genes into several marker groups (Figure 2, right panels). These genes included normally methylated markers (genes methylated in all MEs), common methylation markers (genes methylated in HME, IME, and LME, but unmethylated in NME), intermediate methylation markers (genes methylated in HME and IME, but unmethylated in LME and NME), and high methylation markers (genes specifically methylated in HME). Within IME samples, FAP neoplasms showed significantly lower methylation levels than sporadic CRC in some normally methylated markers, commonly methylated markers, and intermediate markers.

### Confirmation of two subtypes of FAP tumors

**Figure 3:**
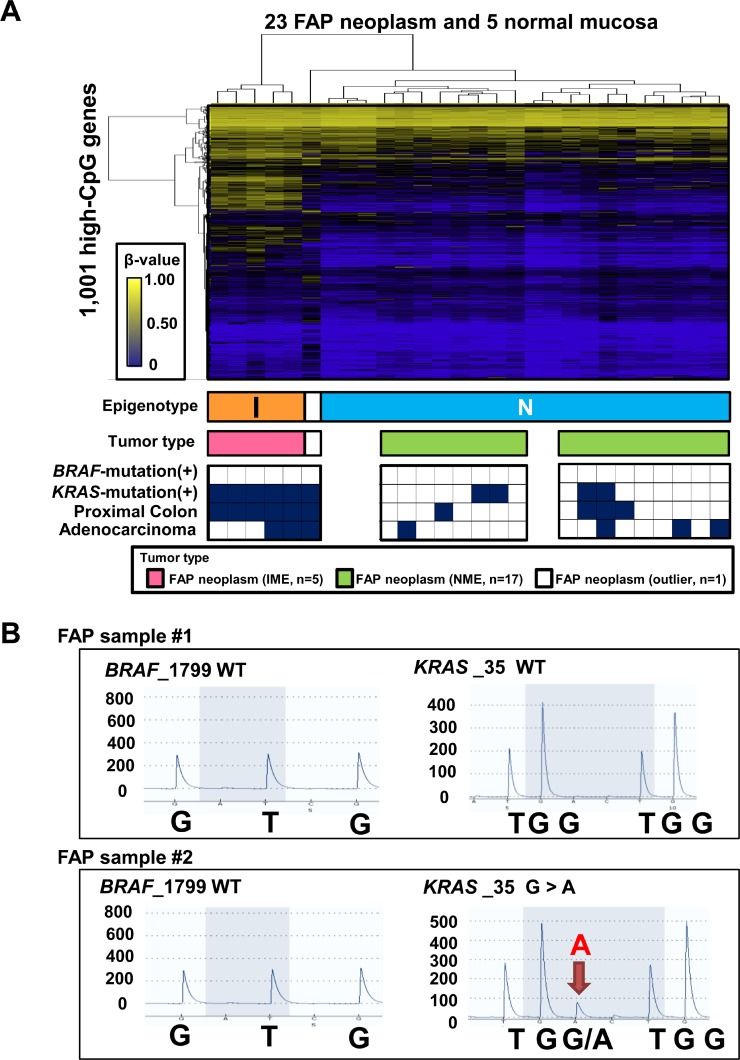
Characterization of 23 FAP neoplasms (**A**) Hierarchical clustering analysis of 23 FAP neoplasm samples and 5 normal mucosa samples without sporadic CRC. *I*, IME; *N*, NME. Bottom closed boxes indicate *BRAF* mutation (+) samples, *KRAS* mutation (+) samples, proximal colon samples, and adenocarcinoma samples. (**B**) Representative pyrograms for mutation analysis of *BRAF* and *KRAS* mutations. *WT*, wild-type.

We performed the two-way hierarchical clustering analysis using the same 1,001 probes only for 23 FAP neoplasms and 5 normal mucosa samples to confirm the two subtypes of FAP neoplasms (Figure 3A). Among the 28 samples, 27 samples were clearly classified into two clusters, whereas one sample was considered as an outlier. The higher methylation cluster (*n* = 5) and lower methylation cluster (*n* = 22) corresponded with IME and NME, respectively, in Figure 2. The five IME neoplasms were all located in the proximal colon, whereas the 17 NME neoplasms were significantly located in the distal colon (*P* = 0.002, Fisher's exact test). There were no significant differences between the benign and malignant properties of neoplasms (three adenoma cases versus two cancer cases in IME and 13 adenoma cases versus four cancer cases in NME; *P* = 0.5).

We then analyzed mutations in *BRAF* and *KRAS* by pyrosequencing (Figure 3B). The five IME tumors were all *KRAS* mutation(+), whereas 13 of 17 NME tumors were *KRAS* mutation(–) (*P* = 0.002; Figure 3A and Supplementary Table 1). These data indicated that IME was significantly correlated with *KRAS* mutations, as reported in sporadic CRC. *BRAF* mutations were not detected in any FAP tumors.

### Extraction of marker genes to characterize four epigenotypes

**Figure 4:**
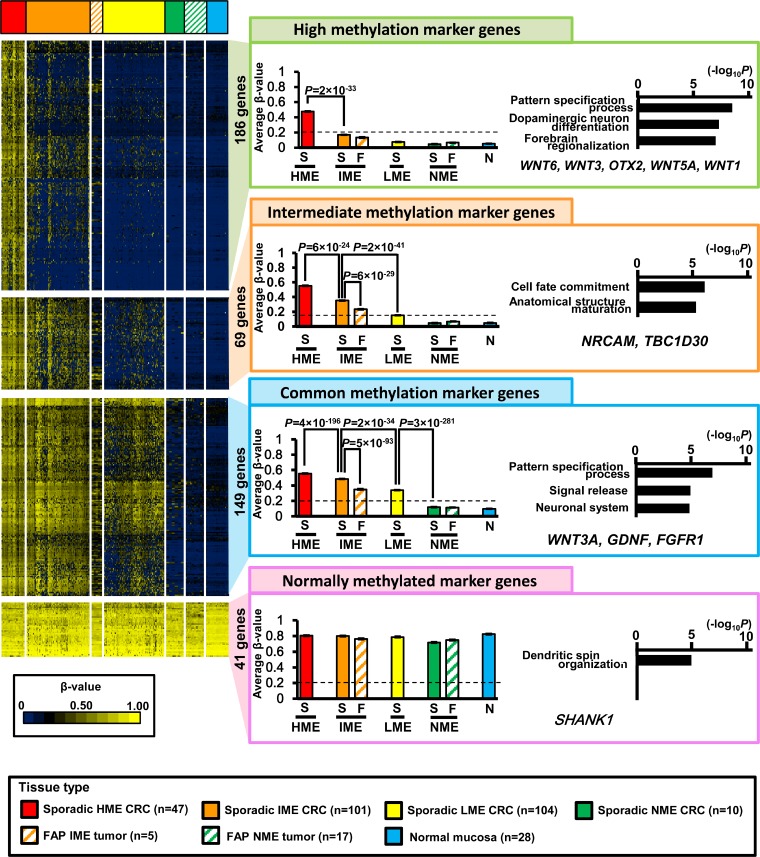
Extraction of marker genes Of all 8,625 high-CpG genes, 186 genes were extracted as high methylation marker genes that were methylated in sporadic HME CRC (average β-value > 0.2), but unmethylated in sporadic IME, LME, and NME CRC (average β-value < 0.2). These genes showed significant enrichment of genes related to pattern specification processes, dopaminergic neuron differentiation, and forebrain regionalization. As intermediate methylation marker genes, 69 genes were identified and showed significant enrichment of genes related to cell fate commitment and anatomical structure maturation. As commonly methylated marker genes, 149 genes were identified and showed significant enrichment of genes related to pattern specification processes, signal release, and neuron systems. As normally methylated marker genes, 41 genes were identified and showed significant enrichment of genes related to dendritic spin organization. *S*, sporadic CRC; *F*, FAP neoplasms; *N*, normal mucosa.

Because hierarchical clustering analysis of gene direction indicated that four marker groups characterized four MEs, we extracted marker genes from the observed 8,625 high-CpG genes (Figure 4).

High methylation marker genes (186 genes) were methylated in HME neoplasms (average β-value ≥ 0.2) but not in IME, LME, and NME (average β-value < 0.2). The average β-value of sporadic HME CRC was significantly higher than that of sporadic IME CRC (0.49 ± 0.00 versus 0.17 ± 0.00, *P* = 2 × 10^−33^, *t*-test). Gene ontology (GO) terms were significantly enriched in genes related to “pattern specification process” and “dopaminergic neuron differentiation” (e.g., *WNT6*, *WNT3*, *OTX2*, *WNT5A*, and *WNT1*).

Intermediate methylation marker genes (69 genes) were methylated in both HME and IME tumors (average β-value ≥ 0.2) but not in LME or NME (average β-value < 0.2). The average β-value of sporadic IME CRC was significantly higher than that of sporadic LME CRC (0.35 ± 0.00 versus 0.15 ± 0.00, *P* = 2 × 10^−41^). The average β-value of sporadic HME CRC was even higher than that of sporadic IME CRC (0.56 ± 0.00 versus 0.35 ± 0.00, *P* = 6 × 10^−24^). GO terms were significantly enriched in genes related to “cell fate commitment” and “anatomical structure maturation” (e.g., *NRCAM* and *TBC1D30*).

Common methylation marker genes (149 genes) were methylated in HME, IME, and LME (average β-value ≥ 0.2), but not in NME (average β-value < 0.2). The average β-value of sporadic LME CRC was significantly higher than that of sporadic NME CRC (0.34 ± 0.00 versus 0.11 ± 0.00, *P* = 3 × 10^−281^), and those of sporadic IME or HME CRC were even higher than that of sporadic LME CRC (0.48 ± 0.00 or 0.55 ± 0.00, respectively). GO terms were significantly enriched in genes related to “pattern specification process”, “signal release”, and “neuronal system” (e.g., *WNT3A*, *GDNF*, and *FGFR1*).

Normally methylated marker genes (41 genes) were methylated in all four MEs (average β-value ≥ 0.2). There were no significant differences among DNA methylation levels in HME, IME, LME, and NME neoplasms.

### Lower methylation levels in FAP tumors

In our analysis of extracted marker genes, aberrant DNA methylation levels of FAP IME neoplasms were significantly lower than those of sporadic IME CRC.

**Figure 5:**
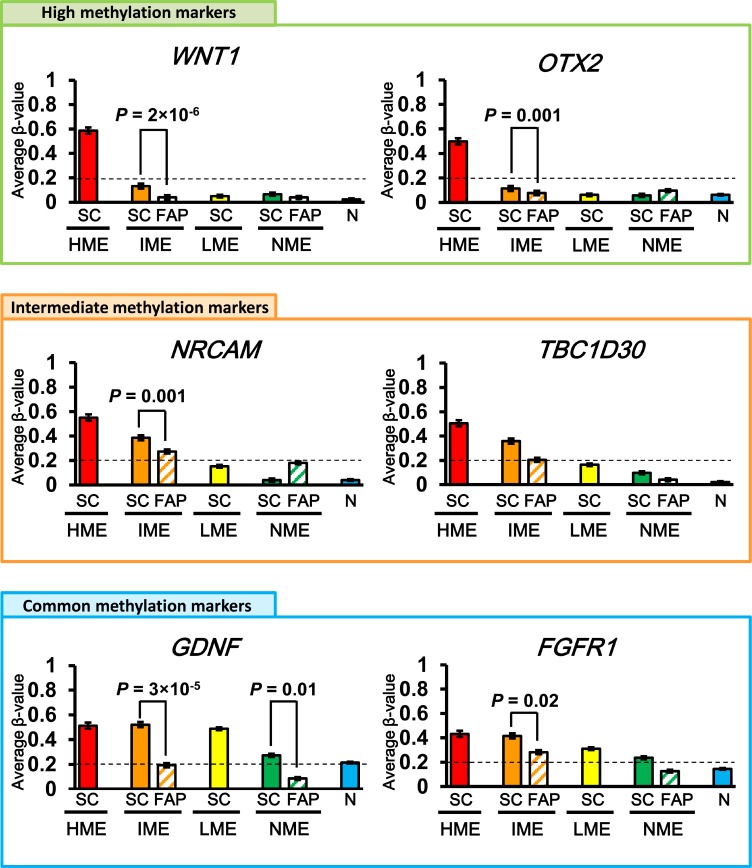
Representative marker genes High methylation marker genes that were methylated in sporadic HME CRC but unmethylated in sporadic IME, LME, and NME CRC (*top*); intermediate methylation marker genes that were methylated in sporadic HME and IME CRC but unmethylated in sporadic LME and NME CRC (*middle*); and commonly methylated marker genes that were methylated in sporadic HME, IME, and LME CRC but unmethylated in sporadic NME CRC (*bottom*) are shown. *SC*, sporadic CRC; *FAP*, FAP neoplasms; *N*, normal mucosa.

In intermediate methylation marker genes, the average β-value of FAP IME neoplasms was 0.22 ± 0.01, which was significantly lower than that of sporadic IME CRC (0.35 ± 0.00, *P* = 6 × 10^−29^, *t*-test) and rather similar to that of sporadic LME CRC (0.15 ± 0.00; Figure 4). Moreover, the β-value of *NRCAM* was significantly lower in FAP IME neoplasms than sporadic IME CRC (*P* = 0.001; Figure 5). The β-value of *TBC1D30* in sporadic IME CRC (0.36 ± 0.00) was higher than that in FAP IME neoplasms (0.21 ± 0.01), which was similar to that in sporadic LME CRC (0.16 ± 0.00; Figure 5).

In common methylation marker genes, FAP IME neoplasms showed a β-value of 0.34 ± 0.01, which was significantly lower than that of sporadic IME CRC (0.48 ± 0.00, *P* = 5 × 10^−93^) and rather similar to that of sporadic LME CRC (0.34 ± 0.00; Figure 4). The β-values of *GDNF* and *FGFR1* were representatively shown, and significantly lower in FAP IME neoplasms than sporadic IME CRC (*P* = 3 × 10^−5^ and *P* = 0.02, respectively; Figure 5).

Although high methylation marker genes should be methylated only in HME CRC samples, and their average β-value in IME CRC samples was < 0.2, β-values were even lower in IME FAP neoplasms than in sporadic IME CRC, e.g., *WNT1* (*P* = 2 × 10^−6^) and *OTX2* (*P* = 0.001), as shown in Figure 5.

### Comparison with adenoma

**Figure 6:**
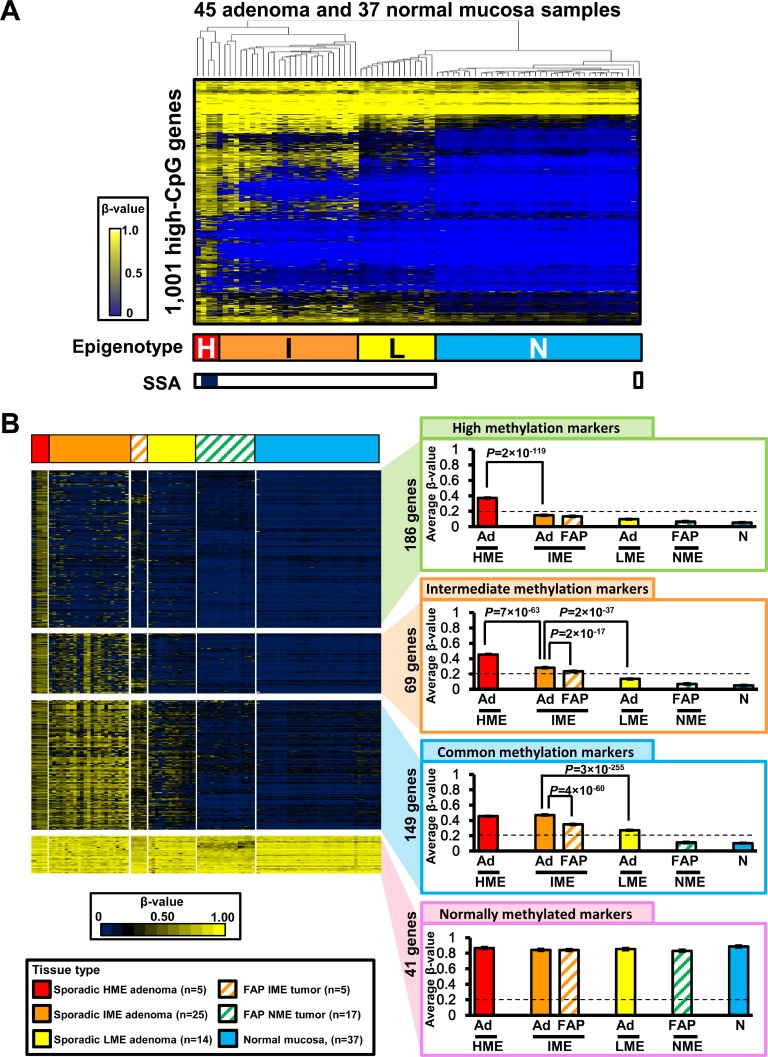
Comparison between FAP neoplasms and sporadic colorectal adenoma (**A**) Hierarchical clustering analysis of sporadic colorectal adenoma and normal mucosa samples. Infinium 450K data of 42 protruded adenoma and three sessile serrated adenoma (SSA) samples were obtained from GSE96540 and GSE48684. The 45 adenoma samples were stratified into four clusters, similar to sporadic CRC. *H*, HME; *I*, IME; *L*, LME; *N*, NME. *Open box*, protruded adenoma; *closed box*, SSA (*bottom*). (**B**) Comparison of methylation levels of methylation marker genes in FAP neoplasms and adenoma samples. *Ad*, sporadic colorectal adenoma; *FAP*, FAP neoplasms; *N*, normal mucosa.

To ensure that the lower methylation level of FAP neoplasms compared with that of sporadic CRC was not related to the presence of adenoma samples in FAP neoplasms, we next analyzed DNA methylation in colorectal adenoma (Figure 6). Infinium 450 k analysis of 45 sporadic colorectal adenoma samples was previously performed (GSE96540 and GSE48684) [17], including 42 protruded adenoma samples and three sessile serrated adenoma (SSA) samples, and these DNA methylation data were analyzed together with 37 normal mucosa samples. In total, 82 samples underwent unsupervised two-way hierarchical clustering analysis and were stratified into four methylation epigenotypes as sporadic CRC (Figure 6A).

All the three SSA samples were classified into HME, together with two protruded adenoma samples. In contrast, 25 sporadic protruded adenoma samples were clustered into IME, 14 adenoma samples were clustered into LME, and one adenoma was clustered into NME together with the 37 normal mucosa samples (Figure 6A).

Similar to the comparison between sporadic CRC and FAP neoplasms in Figure 4, we compared DNA methylation levels of extracted methylation marker genes among adenoma and FAP neoplasm samples in each methylation epigenotype (Figure 6B). Overall, 186 high methylation marker genes were methylated in sporadic HME adenoma samples (average β-value ≥ 0.2) but unmethylated in IME, LME, and NME samples (average β-value < 0.2), and there was significant difference between sporadic HME adenoma and sporadic IME adenoma (*P* = 2 × 10^−119^, *t*-test). In total, 69 intermediate methylation markers were methylated in HME and IME (average β-value ≥ 0.2), but unmethylated in LME and NME (average β-value < 0.2). Sporadic IME adenoma showed significantly higher methylation levels than LME adenoma (β-value 0.28 ± 0.01 versus 0.11 ± 0.00, *P* = 2 × 10^−37^, *t*-test), and sporadic HME adenoma showed even higher methylation levels (β-value 0.46 ± 0.10, *P* = 7 × 10^−63^). Additionally, 149 common methylation marker genes were methylated in HME, IME, and LME (average β-value ≥ 0.2), but unmethylated in NME (average β-value < 0.2), and 41 normally methylated marker genes were all methylated in HME, IME, LME, and NME samples (average β-value ≥ 0.2), including 37 normal mucosa samples (Figure 6B).

Methylation in FAP neoplasms was also lower than that in sporadic colorectal adenoma (Figure 6B). Methylation levels of 69 intermediate and 149 common methylation marker genes were significantly lower in FAP IME neoplasms than in sporadic IME adenoma (β-value: 0.23 ± 0.01 versus 0.28 ± 0.01, *P* = 2 × 10^−17^; and β-value: 0.35 ± 0.01 versus 0.46 ± 0.00, *P* = 4 × 10^−60^, respectively).

## DISCUSSION

There is enormous heterogeneity in sporadic CRC [8, 10, 21]. CRCs vary according to not only clinicopathological features, e.g., location and histology, but also molecular features, e.g., DNA methylation levels, microsatellite instability, chromosomal instability, and the presence of somatic or germline mutations. While these factors may interact with one another, sporadic CRC develops through several different tumorigenic pathways. Although comprehensive molecular characterizations of sporadic colorectal adenoma and cancer have been performed, the detailed molecular features of FAP-associated colorectal neoplasms are still unclear. In this study, we aimed to clarify the presence of multiple methylation epigenotypes in FAP-associated colorectal neoplasms through genome-wide DNA methylation analysis and to elucidate the distinction between FAP-associated and sporadic colorectal neoplasms. Sporadic CRC could be classified into four MEs (HME, IME, LME, and NME); however, FAP-associated neoplasms were classified only into two MEs (IME and NME). FAP IME neoplasms, however, had significantly lower levels of aberrant DNA methylation than sporadic IME colorectal adenoma/cancer, suggesting that the molecular basis of FAP neoplasms differed from that of sporadic colorectal tumorigenesis.

We and others previously classified CRC into three distinct epigenotypes: HME, IME, and LME [8–10]. Although HME and IME were strongly correlated with *BRAF* and *KRAS* mutations, respectively, LME cases were not associated with these oncogenic mutations, suggesting the existence of distinct pathways in colorectal carcinogenesis. In this study, the fourth ME was identified in both FAP-associated and sporadic colorectal neoplasms. Since these neoplasm samples hardly showed aberrant hypermethylation, and clustered with all the normal mucosa samples, this fourth ME was named normal-like ME (NME). The existence of NME in both sporadic protruded adenoma and cancer suggested that a fraction of sporadic CRC could develop without accumulation of aberrant promoter hypermethylation. Interestingly, a major fraction of FAP neoplasms were classified as NME. Because inactivation of *APC* is supposed to occur at an early stage of FAP tumorigenesis, aberrant hypermethylation of genes, especially with regard to negative regulators for WNT signaling, may not be required in the majority of FAP neoplasms, and genetic mutations, e.g. *TP53* mutation, might be required.

*KRAS* mutations were detected in a fraction of FAP neoplasms and were significantly correlated with IME in FAP neoplasms, as observed in sporadic CRC [8–10]. Although most FAP neoplasms develop without requiring aberrant methylation, some FAP neoplasms develop in the presence of both IME and *KRAS* mutations, demonstrating aberrant methylation and oncogene mutations.

Mutations in *APC*, *KRAS*, and *TP53* are well-known genetic alterations, which were demonstrated in the model of adenoma-carcinoma sequence, and *KRAS* mutation plays a major role in the progression from low-grade to high-grade adenoma [22]. Whereas sporadic CRC is known to be stratified into several molecular subtypes with/without these mutations and the tumorigenic pathway is more complicated [8, 10, 21], accumulation of DNA hypermethylation and *KRAS* mutation was demonstrated to be completed by adenoma stage in IME neoplasms [23], in good agreement with adenoma-carcinoma sequence. In the progression from adenoma to intramucosal carcinoma, *TP53* mutation was reportedly required in both *KRAS*-mutation(+) and *KRAS*-mutation(−) neoplasms [23]. *TP53* mutation is also frequently observed in FAP neoplasms, and suggested to play a critical role in FAP tumorigenesis [24, 25]. Further studies are necessary to clarify at which stage of tumorigenesis *TP53* mutation is required in each subtype of FAP neoplasm.

*BRAF* mutations were not detected in FAP neoplasms, and HME was also not observed in FAP neoplasms. These data are consistent with previous reports demonstrating that sporadic HME CRC was strongly correlated with *BRAF* mutations [8, 26] and that SSA was a precursor for sporadic HME CRC showing both HME and *BRAF* mutations [23]. Thus, the serrated pathway of sporadic CRC may not be involved in FAP tumorigenesis.

Interestingly, the methylation levels of intermediate methylation and commonly methylated markers were lower in IME FAP neoplasms than sporadic IME CRC. These genes showing lower methylation included *WNT1*, *OTX2*, *GDNF*, and *FGFR1*. The proposed mechanisms for the oncogenic function of *OTX2* include transactivation of cell cycle genes and induction of the *MYC* oncogene, which play key roles in tumor maintenance in some medulloblastomas [27, 28]. *MYC* is upregulated by WNT signaling [29]. Moreover, although WNT signaling is activated by *APC* mutations in FAP neoplasms, reduced inactivation of *OTX2* and activation of *MYC* may contribute to tumor development in FAP.

*GDNF* has been shown to enhance the migration of colon cancer cells by increasing vascular endothelial growth factor (VEGF)/VEGF receptor interactions, which are mainly regulated by the p38, phosphatidylinositol 3-kinase/Akt, and hypoxia-inducible factor 1α signaling pathways [30]. *GDNF* has been reported to be hypermethylated in colorectal carcinogenesis [31, 32]; however, reduced inactivation of *GDNF* may also contribute to FAP tumorigenesis. *FGFR1* was reported to be highly expressed in colorectal adenoma and cancer, and activating mutations and overexpression of *FGFR1* have been shown to trigger the development of various cancers, e.g., breast, bladder, ovarian cancer, renal cell, and squamous cell lung cancers [33–35]. These findings suggest that lower methylation of these oncogenic genes in FAP-associated IME neoplasms might perhaps contribute to the early development of adenoma/cancer in FAP. Further studies of these genes and roles of their reduced methylation should be conducted to fully clarify the tumorigenic mechanisms of FAP neoplasms.

Limitation of this study is that FAP neoplasm samples were obtained from only two patients; thus the current results may not be generalizable to all the FAP neoplasms. Although it should be necessary to conduct comprehensive genome-wide analysis using samples from more FAP patients, our study clearly demonstrated that there are at least two DNA methylation epigenotypes in FAP neoplasms, and that FAP neoplasms involve different molecular features from sporadic CRC/adenoma, e.g. lower levels of aberrant DNA methylation.

In summary, sporadic colorectal adenoma and cancer were classified into four MEs (HME, IME, LME, and NME), whereas FAP neoplasms were classified into at least two subtypes (IME and NME). The significantly lower levels of aberrant methylation observed in FAP-associated IME neoplasms compared with that in sporadic IME CRC/adenoma suggested that aberrant DNA methylation may contribute to tumorigenesis of FAP in a manner different from that of sporadic CRC.

## MATERIALS AND METHODS

### Clinical samples

A total of 23 colorectal neoplasm samples (16 adenoma samples and seven cancer samples) were obtained from two patients with FAP who underwent operation at Chiba University Hospital with written informed consent. The germline mutations of *APC* and other clinicopathological characteristics of the samples of the two patients are shown in Supplementary Table 1. Colorectal samples were fixed with 10% formalin and then embedded in paraffin. The FFPE samples were sectioned into 10-μm-thick slices using a paraffin sectioning method. FAP samples were microscopically examined for determination of neoplastic cell contents by two independent pathologists and were dissected to enrich neoplastic cells when necessary. For molecular analysis, 23 FAP samples that contained at least 40% neoplastic cells were used. When necessary, samples were subjected to laser microdissection using an ArcturusXT instrument (Life Technologies, Carlsbad, CA, USA) to enrich tumor cells. The DNA was extracted using a QIAamp DNA FFPE Tissue Kit (Qiagen, Hilden, Germany). This study was approved by the Ethics Committee of Chiba University.

### Quality check of FFPE DNAs

The quality of DNA from FFPE blocks was checked using an Infinium FFPE QC Kit (Illumina, San Diego, CA, USA) by triplicate quantitative polymerase chain reaction using 1 ng DNA. The ΔCq was calculated by subtracting the average Cq value of the interrogated sample from the Cq value of the standard sample provided by the manufacturer. All 23 FFPE samples showed ΔCq values of less than 5, which is the recommended threshold for suitability for FFPE restoration.

### Bisulfite conversion

Bisulfite conversion was performed using a Zymo EZ DNA Methylation Kit (Zymo Research, Irvine, CA, USA) with 250 ng genomic DNA for each sample following the manufacturer's instructions for Infinium assays.

### Infinium assays

The Infinium HumanMethylation450 BeadChip (Illumina) contains approximately 485,000 individual CpG sites covering 99% of RefSeq genes with an average of 17 CpG sites/gene. For each CpG site, the β-value, ranging from 0.00 to 1.00, was measured by a methylated probe relative to the sum of both methylated and unmethylated probes. Whole-genome amplification, labeling, hybridization, and scanning were performed according to the manufacturer's protocols. Infinium analysis was performed against 23 FAP samples, and Infinium data were submitted to the NCBI Gene Expression Omnibus (GEO) database under accession numbers GSE109507 (GSM2944754–GSM2944776).

For analysis of DNA methylation at the promoter regions based on Infinium data, a single probe nearest to the transcription start site was selected when multiple probes were designed for one promoter region. The CpG score of the 500-bp region around each probe (***±***250 bp) was calculated based on previous reports [36–38], and genes containing probes with CpG scores of more than 0.48 in the promoter regions were defined as high-CpG genes.

### Mutation analysis

FAP samples were tested for mutations in *BRAF* (1799) and *KRAS* (34, 35, 37, and 38) by pyrosequencing using PyroMark Gold Q96 reagents (Qiagen) and a PyroMark Q96 ID instrument, as previously reported [39] (Figure 3B). Primer sequences are described in Supplementary Table 2. Pyrogram outputs were analyzed with PyroMark Q96 ID Software (Qiagen) using the allele quantification mode. The cut-off value for positive results of mutations was set at 20% on the sequencer and was based on the tumor cell content (≥40%).

### Public datasets

For validation, we used independent Infinium datasets supplied by TCGA, including 297 sporadic CRC samples and 37 normal colorectal mucosa samples (https://tcga-data.nci.nih.gov/docs/publications/tcga/), and those from other datasets, including 42 sporadic colorectal protruded adenomas (GSE48684) and three SSAs (GSE96540) [17].

### Comparison between frozen and FFPE samples

To avoid obtaining different β-values for DNA samples from frozen and FFPE tissues for some probes, three colon tumor samples were cut into two pieces; one was frozen immediately after surgical resection, and the other was treated by the FFPE procedure (Figure 1). Three pairs of frozen and FFPE samples (from three tumor samples) were analyzed by Infinium. Probes showing differences in β-values between frozen and FFPE samples of less than 0.1 for all three pairs were extracted and used for analysis of frozen and FFPE samples together.

### GO analysis

Gene annotation enrichment analysis was conducted based on “biologic process”, “cellular component”, and “molecular function” categories using the Functional Annotation tool of Metascape (http://metascape.org/gp/index.html#/main/step1) [40].

### Statistical analysis

Unsupervised two-way hierarchical clustering was performed using *R* software (https://www.r-project.org/). Unsupervised two-way hierarchical clustering was done based on standard correlation and average linkage clustering algorism in sample direction, and City-block distance and complete linkage clustering algorism in marker direction. Associations between clinicopathological factors and DNA methylation were analyzed using Fisher's exact test and Student's *t*-tests.

## SUPPLEMENTARY MATERIALS FIGURES AND TABLES



## References

[1] GBD 2013 Mortality and Causes of Death Collaborators (2015). Global, regional, and national age-sex specific all-cause and cause-specific mortality for 240 causes of death, 1990–2013: a systematic analysis for the Global Burden of Disease Study 2013. Lancet.

[2] Vogelstein B, Papadopoulos N, Velculescu VE, Zhou S, Diaz LA, Kinzler KW (2013). Cancer genome landscapes. Science.

[3] Lao VV, Grady WM (2011). Epigenetics and colorectal cancer. Nat Rev Gastroenterol Hepatol.

[4] Grady W.M, Carethers JM (2008). Genomic and epigenetic instability in colorectal cancer pathogenesis. Gastroenterology.

[5] Jones PA, Baylin SB (2007). The epigenomics of cancer. Cell.

[6] Burrell RA, McGranahan N, Bartek J, Swanton C (2013). The causes and consequences of genetic heterogeneity in cancer evolution. Nature.

[7] Kaneda A, Matsusaka K, Sakai E, Funata S (2014). DNA methylation accumulation and its predetermination of future cancer phenotypes. J Biochem.

[8] Yagi K, Akagi K, Hayashi H, Nagae G, Tsuji S, Isagawa T, Midorikawa Y, Nishimura Y, Sakamoto H, Seto Y, Aburatani H, Kaneda A (2010). Three DNA methylation epigenotypes in human colorectal cancer. Clin Cancer Res.

[9] Shen L, Toyota M, Kondo Y, Lin E, Zhang L, Guo Y, Hernandez NS, Chen X, Ahmed S, Konishi K, Hamilton SR, Issa JP (2007). Integrated genetic and epigenetic analysis identifies three different subclasses of colon cancer. Proc Natl Acad Sci U S A.

[10] Hinoue T, Weisenberger DJ, Lange CP, Shen H, Byun HM, Van Den Berg D, Malik S, Pan F, Noushmehr H, van Dijk CM, Tollenaar RA, Laird PW (2012). Genome-scale analysis of aberrant DNA methylation in colorectal cancer. Genome Res.

[11] Jiang SS, Li JJ, Li Y, He LJ, Wang QJ, Weng DS, Pan K, Liu Q, Zhao JJ, Pan QZ, Zhang XF, Tang Y, Chen CL (2015). A novel pathogenic germline mutation in the adenomatous polyposis coli gene in a Chinese family with familial adenomatous coli. Oncotarget.

[12] Galiatsatos P, Foulkes WD (2006). Familial adenomatous polyposis. Am J Gastroenterol.

[13] Will OC, Leedham SJ, Elia G, Phillips RK, Clark SK, Tomlinson IP (2010). Location in the large bowel influences the APC mutations observed in FAP adenomas. Fam Cancer.

[14] Bisgaard ML, Ripa R, Knudsen AL, Bulow S (2004). Familial adenomatous polyposis patients without an identified APC germline mutation have a severe phenotype. Gut.

[15] Wynter CV, Kambara T, Walsh MD, Leggett BA, Young J, Jass JR (2006). DNA methylation patterns in adenomas from FAP, multiple adenoma and sporadic colorectal carcinoma patients. Int J Cancer.

[16] Takane K, Matsusaka K, Ota S, Fukuyo M, Yue Y, Nishimura M, Sakai E, Matsushita K, Miyauchi H, Aburatani H, Nakatani Y, Takayama T, Matsubara H (2016). Two subtypes of colorectal tumor with distinct molecular features in familial adenomatous polyposis. Oncotarget.

[17] Luo Y, Wong CJ, Kaz AM, Dzieciatkowski S, Carter KT, Morris SM, Wang J, Willis JE, Makar KW, Ulrich CM, Lutterbaugh JD, Shrubsole MJ, Zheng W (2014). Differences in DNA methylation signatures reveal multiple pathways of progression from adenoma to colorectal cancer. Gastroenterology.

[18] Srinivasan M, Sedmak D, Jewell S (2002). Effect of fixatives and tissue processing on the content and integrity of nucleic acids. Am J Pathol.

[19] de Ruijter TC, de Hoon JP, Slaats J, de Vries B, Janssen MJ, van Wezel T, Aarts MJ, van Engeland M, Tjan-Heijnen VC, Van Neste L, Veeck J (2015). Formalin-fixed, paraffin-embedded (FFPE) tissue epigenomics using Infinium HumanMethylation450 BeadChip assays. Lab Invest.

[20] Moran S, Vizoso M, Martinez-Cardús A, Gomez A, Matías-Guiu X, Chiavenna SM, Fernandez AG, Esteller M (2014). Validation of DNA methylation profiling in formalin-fixed paraffin-embedded samples using the Infinium HumanMethylation450 Microarray. Epigenetics.

[21] Cancer Genome Atlas Network (2012). Comprehensive molecular characterization of human colon and rectal cancer. Nature.

[22] Vogelstein B, Fearon ER, Hamilton SR, Kern SE, Preisinger AC, Leppert M, Nakamura Y, White R, Smits AM, Bos JL (1988). Genetic alterations during colorectal-tumor development. N Engl J Med.

[23] Sakai E, Fukuyo M, Ohata K, Matsusaka K, Doi N, Mano Y, Takane K, Abe H, Yagi K, Matsuhashi N, Fukushima J, Fukayama M, Akagi K (2016). Genetic and epigenetic aberrations occurring in colorectal tumors associated with serrated pathway. Int J Cancer.

[24] Kikuchi-Yanoshita R, Konishi M, Ito S, Seki M, Tanaka K, Maeda Y, Iino H, Fukayama M, Koike M, Mori T, Sakuraba H, Fukunari H, Iwama T (1992). Genetic changes of both p53 alleles associated with the conversion from colorectal adenoma to early carcinoma in familial adenomatous polyposis and non-familial adenomatous polyposis patients. Cancer Res.

[25] Shirasawa S, Urabe K, Yanagawa Y, Toshitani K, Iwama T, Sasazuki T (1991). p53 gene mutations in colorectal tumors from patients with familial polyposis coli. Cancer Res.

[26] Weisenberger DJ, Siegmund KD, Campan M, Young J, Long TI, Faasse MA, Kang GH, Widschwendter M, Weener D, Buchanan D, Koh H, Simms L, Barker M (2006). CpG island methylator phenotype underlies sporadic microsatellite instability and is tightly associated with BRAF mutation in colorectal cancer. Nat Genet.

[27] Adamson DC, Shi Q, Wortham M, Northcott PA, Di C, Duncan CG, Li J, McLendon RE, Bigner DD, Taylor MD, Yan H (2010). OTX2 is critical for the maintenance and progression of Shh-independent medulloblastomas. Cancer Res.

[28] Bunt J, Hasselt NE, Zwijnenburg DA, Hamdi M, Koster J, Versteeg R, Kool M (2012). OTX2 directly activates cell cycle genes and inhibits differentiation in medulloblastoma cells. Int J Cancer.

[29] Dominguez-Brauer C, Khatun R, Elia AJ, Thu KL, Ramachandran P, Baniasadi SP, Hao Z, Jones LD, Haight J, Sheng Y, Mak TW (2017). E3 ubiquitin ligase Mule targets beta-catenin under conditions of hyperactive Wnt signaling. Proc Natl Acad Sci U S A.

[30] Huang SM, Chen TS, Chiu CM, Chang LK, Liao KF, Tan HM, Yeh WL, Chang GR, Wang MY, Lu DY (2013). GDNF increases cell motility in human colon cancer through VEGF-VEGFR1 interaction. Endocr Relat Cancer.

[31] Sambuudash O, Kim HS, Cho MY (2017). Lack of Aberrant Methylation in an Adjacent Area of Left-Sided Colorectal Cancer. Yonsei Med J.

[32] Cavel O, Shomron O, Shabtay A, Vital J, Trejo-Leider L, Weizman N, Krelin Y, Fong Y, Wong RJ, Amit M, Gil Z (2012). Endoneurial macrophages induce perineural invasion of pancreatic cancer cells by secretion of GDNF and activation of RET tyrosine kinase receptor. Cancer Res.

[33] Dutt A, Ramos AH, Hammerman PS, Mermel C, Cho J, Sharifnia T, Chande A, Tanaka KE, Stransky N, Greulich H, Gray NS, Meyerson M (2011). Inhibitor-sensitive FGFR1 amplification in human non-small cell lung cancer. PLoS One.

[34] Weiss J, Sos ML, Seidel D, Peifer M, Zander T, Heuckmann JM, Ullrich RT, Menon R, Maier S, Soltermann A, Moch H, Wagener P, Fischer F (2010). Frequent and focal FGFR1 amplification associates with therapeutically tractable FGFR1 dependency in squamous cell lung cancer. Sci Transl Med.

[35] Liang G, Liu Z, Wu J, Cai Y, Li X (2012). Anticancer molecules targeting fibroblast growth factor receptors. Trends Pharmacol Sci.

[36] Weber M, Hellmann I, Stadler MB, Ramos L, Paabo S, Rebhan M, Schubeler D (2007). Distribution, silencing potential and evolutionary impact of promoter DNA methylation in the human genome. Nat Genet.

[37] Matsusaka K, Kaneda A, Nagae G, Ushiku T, Kikuchi Y, Hino R, Uozaki H, Seto Y, Takada K, Aburatani H, Fukayama M (2011). Classification of Epstein-Barr virus-positive gastric cancers by definition of DNA methylation epigenotypes. Cancer Res.

[38] Nakagawa T, Matsusaka K, Misawa K, Ota S, Takane K, Fukuyo M, Rahmutulla B, Shinohara KI, Kunii N, Sakurai D, Hanazawa T, Matsubara H, Nakatani Y (2017). Frequent promoter hypermethylation associated with human papillomavirus infection in pharyngeal cancer. Cancer Lett.

[39] Seymour MT, Brown SR, Middleton G, Maughan T, Richman S, Gwyther S, Lowe C, Seligmann JF, Wadsley J, Maisey N, Chau I, Hill M, Dawson L (2013). Panitumumab and irinotecan versus irinotecan alone for patients with KRAS wild-type, fluorouracil-resistant advanced colorectal cancer (PICCOLO): a prospectively stratified randomised trial. Lancet Oncol.

[40] Tripathi S, Pohl MO, Zhou Y, Rodriguez-Frandsen A, Wang G, Stein DA, Moulton HM, DeJesus P, Che J, Mulder LC, Yángüez E, Andenmatten D, Pache L (2015). Meta- and Orthogonal Integration of Influenza “OMICs” Data Defines a Role for UBR4 in Virus Budding. Cell Host Microbe.

