# Supplementary material for: The frequency of promoter DNA hypermethylation is decreased in colorectal neoplasms of familial adenomatous polyposis
Source: Oncotarget. 2018 Aug 24;9(66):32653–66. doi: 10.18632/oncotarget.25987 (PMC6135695; doi:10.18632/oncotarget.25987)
Supplement: Supplementary file 1 [file oncotarget-09-32653-s001.pdf]

The frequency of promoter DNA hypermethylation is decreased in colorectal neoplasms of familial adenomatous polyposis

SUPPLEMENTARY MATERIALS

Supplementary Table 1: Clinicopathologic characteristics of FAP neoplasm samples

Sample	Sex	Age (year)	*APC* mutation	Pathology	*KRAS* mutation	Methylaton epigenotype	Tumor location
1–1	Male	29	L540X	Tubular Ad, low grade	−	NME	Distal
1–2	Male	29	L540X	Tubular Ad, low grade	−	NME	Distal
1–3	Male	29	L540X	Tubular Ad, low grade	−	NME	Distal
1–4	Male	29	L540X	Tubular Ad, high grade	+	NME	Distal
1–5	Male	29	L540X	Ca	−	NME	Distal
1–6	Male	29	L540X	Ca	−	NME	Distal
1–7	Male	29	L540X	Tubular Ad, low grade	+	NME	Distal
1–8	Male	29	L540X	Tubular Ad, low grade	−	NME	Distal
1–9	Male	29	L540X	Tubular Ad, low grade	+	NME	Proximal
1–10	Male	29	L540X	Tubular Ad, low grade	−	NME	Proximal
1–11	Male	29	L540X	Tubular Ad, low grade	–	NME	Distal
1–12	Male	29	L540X	Tubular Ad, high grade	–	NME	Distal
1–13	Male	29	L540X	Ca	+	NME	Proximal
1–14	Male	29	L540X	Tubular Ad, low grade	–	NME	Proximal
1–15	Male	29	L540X	Ca	+	outlier	Proximal
2–1	Female	50	R216X	Ca	−	NME	Distal
2–2	Female	50	R216X	Tubular Ad, low grade	−	NME	Distal
2–3	Female	50	R216X	Tubular Ad, low grade	−	NME	Distal
2–4	Female	50	R216X	Tubular Ad, low grade	+	IME	Proximal
2–5	Female	50	R216X	Ca	+	IME	Proximal
2–6	Female	50	R216X	Ca	+	IME	Proximal
2–7	Female	50	R216X	Tubular Ad, low grade	+	IME	Proximal
2–8	Female	50	R216X	Tubular Ad, low grade	+	IME	Proximal

Abbreviations: Ad, adenoma. Ca, adenocarcinoma.

Supplementary Table 2: Pyrosequencing primers for mutation analysis

Primer sequences	Anneal	Product	Location (hg38)
KRAS (codons 12 & 13)			
Fwd: GGCCTGCTGAAAATGACTGA	58° C	80 bp	chr12:25,363,705-25,363,784
Rev*: AGCTGTATCGTCAAGGCACTCT			
Seq: AAACTTGTGGTAGTTGGA			
BRAF (codon 600)			
Fwd: TGAAGACCTCACAGTAAAAATAGG	58° C	91 bp	chr7:140,605,198-140,605,288
Rev*: TCCAGACAACTGTTCAAACTGAT			
Seq: TGATTTTGGTCTAGCTACA			

Abbreviations: Fwd: forward primer. Rev: reverse primer. Seq: sequence primer. *Primers with 5′-biotin tag. After PCR amplification using the forward and reverse primers, pyrosequencing was performed using the sequence primer, as previously reported [1].

REFERENCES

1. Seymour MT, Brown SR, Middleton G, Maughan T, Richman S, Gwyther S, Lowe C, Seligmann JF, Wadsley J, Maisey N, Chau I, Hill M, Dawson L, et al. Panitumumab and irinotecan versus irinotecan alone for patients with KRAS wild-type, fluorouracil-resistant advanced colorectal cancer (PICCOLO): a prospectively stratified randomised trial. Lancet Oncol. 2013; 14:749–759.
